# ERN GENTURIS guidelines on constitutional mismatch repair deficiency diagnosis, genetic counselling, surveillance, quality of life, and clinical management

**DOI:** 10.1038/s41431-024-01708-6

**Published:** 2024-10-17

**Authors:** Chrystelle Colas, Léa Guerrini-Rousseau, Manon Suerink, Richard Gallon, Christian P. Kratz, Éloïse Ayuso, ERN GENTURIS CMMRD Guideline Group, Chrystelle Colas, Léa Guerrini-Rousseau, Manon Suerink, Richard Gallon, Christian P. Kratz, Éloïse Ayuso, Felipe Andreiuolo, Amedeo A. Azizi, Kevin Beccaria, Birgit Burkhardt, Beatrice Claret, Volodia Dangouloff-ros, Youenn Drouet, Marjolijn C. J. Jongmans, Mariëtte van Kouwen, Clara Ruiz-Ponte, Magali Svrcek, Laurence Brugières, Katharina Wimmer, Laurence Brugières, Katharina Wimmer

**Affiliations:** 1 Institut Curie, Paris, France; 2 Gustave Roussy Cancer Center, Villejuif, France; 3 Department of Clinical Genetics, Leiden University Medical Centre, Leiden, the Netherlands; 4 Translational and Clinical Research Institute, Newcastle University, Newcastle upon Tyne, UK; 5 Pediatric Hematology and Oncology, Hannover Medical School, Hannover, Germany; 6 Patient representative, Bordeaux, France; 7 Institute of Human Genetics, Medical University of Innsbruck, Innsbruck, Austria; 8 Department of Pathology Rede D’Or, Rio de Janeiro, RJ Brazil; 9 D’Or Institute for Research and Education (IDOR), Rio de Janeiro, RJ Brazil; 10 Instituto Estadual do Cérebro Paulo Niemeyer, Rio de Janeiro, RJ Brazil; 11 Division of Neonatology, Pediatric Intensive Care and Neuropediatrics, Department of Pediatrics and Adolescent Medicine, Medical University of Vienna, Vienna, Austria; 12 Necker – Enfants Malades hospital, Paris Cité University, Paris, France; 13 University Hospital Muenster, Muenster, Germany; 14 Centre Léon Bérard, Lyon, France; 15 Université de Lyon, Villeurbanne, France; 16 Princess Máxima Center, Utrecht, The Netherlands; 17 University Medical Center Utrecht, Utrecht, The Netherlands; 18 Radboud university medical center, Nijmegen, The Netherlands; 19 Fundacion Publica Galega de Medicina Xenomica, Instituto de Investigacion Sanitaria de Santiago, Grupo de Medicina Xenomica-USC, Centro de Investigación Biomédica en Red de Enfermedades Raras, Santiago de Compostela, Spain; 20 Sorbonne Universite, APHP, Saint-Antoine hospital, Paris, France

**Keywords:** Paediatric cancer, Preventive medicine

## Abstract

Constitutional mismatch repair deficiency (CMMRD), first described 25 years ago, confers an extremely high and lifelong cancer risk, including haematologic, brain, and gastrointestinal tract malignancies, and is associated with several non-neoplastic features. Our understanding of this condition has improved and novel assays to assist CMMRD diagnosis have been developed. Surveillance protocols need adjustment taking into account recent observational prospective studies assessing their effectiveness. Response to immune checkpoint inhibitors and the effectiveness and toxicity of other treatments have been described. An update and merging of the different guidelines on diagnosis and clinical management of CMMRD into one comprehensive guideline was needed. Seventy-two expert members of the European Reference Network GENTURIS and/or the European care for CMMRD consortium and one patient representative developed recommendations for CMMRD diagnosis, genetic counselling, surveillance, quality of life, and clinical management based on a systematic literature search and comprehensive literature review and a modified Delphi process. Recommendations for the diagnosis of CMMRD provide testing criteria, propose strategies for CMMRD testing, and define CMMRD diagnostic criteria. Recommendations for surveillance cover each CMMRD-associated tumour type and contain information on starting age, frequency, and surveillance modality. Recommendations for clinical management cover cancer treatment, management of benign tumours or non-neoplastic features, and chemoprevention. Recommendations also address genetic counselling and quality of life. Based on existing guidelines and currently available data, we present 82 recommendations to improve and standardise the care of CMMRD patients in Europe. These recommendations are not meant to be prescriptive and may be adjusted based on individual decisions.

## Introduction

A major role of the DNA mismatch repair (MMR) system is to correct replication errors that escape proofreading by the replicative DNA polymerases Pol ε and Pol δ. MMR deficiency, resulting from inactivation of one of four MMR genes (*MLH1*, MIM# 120436; *MSH2*, MIM# 609309; *MSH6*, MIM# 600678; *PMS2*, MIM# 600259), leads to an increased mutation rate and is frequently found in several types of cancer [1]. Constitutional (hereafter referred to as germline) pathogenic variants (PV) in one of these four genes are associated with an increased cancer risk involving multiple organs. Heterozygous germline MMR gene PV cause Lynch syndrome (LS), an autosomal dominant, adult-onset cancer syndrome mainly predisposing to colorectal and endometrial carcinoma, as well as other cancers at a lower frequency [2]. In LS cancers, MMR deficiency results from somatic inactivation of the second allele. Constitutional MMR deficiency (CMMRD) is caused by biallelic germline PV in one of the MMR genes. Individuals with CMMRD, therefore, lack a functional DNA MMR system in all tissues, which is critical for the maintenance of genomic stability by repair of DNA replication errors. An increased constitutional mutation rate is proposed to drive tumourigenesis in several organ systems. Since the first two reports of CMMRD in 1999 [3, 4], well over 200 children and young adults with this recessively inherited condition have been reported, showing it to be a distinct early-onset cancer predisposition syndrome (OMIM #276300).

Although attenuated forms of CMMRD exist [5–7], the cancer risk in CMMRD is likely the highest among cancer predisposition syndromes, with cancer development occurring as early as the first year of life [8]. According to our review of published patients, as well as data from the C4CMMRD database and a recently published patient cohort [9] (patients may partly overlap), 80–90% of CMMRD patients develop their first tumour before the age of 18 years and about half of the patients before the age of 10 years. The known spectrum of CMMRD-associated cancers is broad and, in essence, any cancer type could be caused by CMMRD. The three most frequent cancer groups are (i) haematological malignancies diagnosed in ~40% of patients, with T-cell lymphoblastic lymphoma being most prevalent, (ii) malignant brain tumours in ~55% of patients, most frequently high-grade gliomas, and, (iii) colorectal carcinomas and other LS-associated tumours in ~50% of patients [10]. Most patients develop digestive tract adenoma often with high-grade dysplasia or (oligo-)polyposis in the second decade of life. Tumour types less frequently seen in CMMRD include sarcoma, neuroblastoma, and nephroblastoma [11–15].

CMMRD is also associated with distinctive non-neoplastic manifestations. The most prevalent non-neoplastic features are café-au-lait maculae reminiscent of neurofibromatosis type 1 (NF1), other hypo- and hyper-pigmented skin patches, and multiple brain developmental venous anomalies [16, 17]. The MMR system is also involved in immunoglobulin class-switch recombination and in somatic hypermutation, two processes needed for B cell maturation and for diversification and specification of the immunoglobulin repertoire. Its deficiency may result in IgG2/4 subclass deficiency, IgA deficiency, or - rarely - more severe phenotypes [18].

The estimated birth incidence of CMMRD, supported by empirical data [19], is approximately one in a million if parents are not related [20]. The prevalence might be substantially higher in populations with founder mutations and/or a high rate of parental consanguinity, with approximately half of CMMRD cases being homozygous for an MMR PV [13]. Over 60% of CMMRD cases have biallelic *PMS2* PV, over 20% biallelic *MSH6* PV, and less than 20% biallelic PV in either *MLH1* or *MSH2* [9, 15]. These numbers reflect the estimated population prevalence of heterozygous PV in these four MMR genes, in which *PMS2* and *MSH6* are about 2.5–4 times more prevalent than PV in *MLH1* and *MSH2* [21]. The substantially lower penetrance of monoallelic *MSH6* and especially *PMS2* PV than that of *MSH2* and *MLH1* PV means CMMRD patients often lack a family history of LS-associated cancers [2, 13].

A diagnosis of CMMRD requires germline genetic testing to identify the causative MMR PV. To provide clear criteria to select childhood or adolescent cancer patients for CMMRD testing, the European consortium Care for CMMRD (C4CMMRD) formulated a scoring system from the cancer type, additional non-malignant neoplasia, CMMRD-associated non-neoplastic features, and family history [15]. As a rare differential diagnosis, CMMRD testing may also be indicated in children without cancer who are suspected to have sporadic NF1 or Legius syndrome but in whom no germline *NF1* or *SPRED1* PV has been identified by comprehensive testing [19, 20]. Several ancillary tests have been developed that assess constitutional loss of MMR function, the pathomechanism underlying CMMRD, and are used to confirm CMMRD in cases where genetic testing renders an inconclusive result [22–28].

CMMRD patients need to be subjected to extensive surveillance due to the high cancer risk and broad tumour spectrum. Several surveillance protocols have been published [8, 11, 29]. In observational prospective studies, these protocols have proven to be effective for brain and digestive tract tumours, but not for haematological malignancies [12, 30].

CMMRD cancers are inherently MMR deficient and this shapes tumour molecular pathology. MMR deficient cancers have increased tumour mutation burden (TMB) and microsatellite instability (MSI). They are frequently classified as hypermutated, which is typically defined as a TMB ≥ 10 mutations per megabase. Mainly in CMMRD brain tumours, but also other tumours, it is common to find concurrent polymerase proofreading deficiency caused by missense PV in the exonuclease domains of replicative DNA polymerases Pol ε or Pol δ [31]. This results in ultramutated tumours with TMB ≥ 100 mutations per megabase, specific mutational signatures, and MSI that is not detectable by classical fragment length analysis-based MSI testing, but by the more sensitive methods that have been developed for MSI testing in constitutional DNA of CMMRD patients [23, 31–33]. Hypermutated and MMR deficient tumours are associated with translation of coding variants producing tumour-specific, immunogenic neoantigens that render the tumours responsive to immune checkpoint inhibitors (ICI) [34]. It follows that ICI are a promising drug class in the treatment of cancer patients with CMMRD, with clinical responses being observed in gastrointestinal and brain tumours [35–37]. MMR deficiency also confers therapy-resistance to tumours, in particular against chemotherapies that rely on functional MMR for their mechanism of action. The inefficacy of temozolomide to treat patients with MMR deficient brain tumours is of particular note for CMMRD patients [38].

## Scope of the guidelines

Existing guidelines for CMMRD diagnosis and cancer surveillance were established by two expert groups, the European consortium C4CMMRD and the International Replication Repair Deficiency Consortium (IRRDC) along with collaborating health care organizations [8, 11, 15, 20, 29, 39]. Recent developments regarding the diagnosis of CMMRD, in particular improved understanding of the CMMRD clinical phenotype and the development of reliable and relatively low cost ancillary assays to complement genetic testing, and regarding the efficacy of cancer surveillance protocols require up-to-date guidelines. Moreover, CMMRD healthcare practice varies and professional guidelines on genetic counselling, quality of life, and cancer treatment are so far lacking. Therefore, based on our current knowledge, ERN GENTURIS and C4CMMRD combined efforts to update the different guidelines on diagnosis and surveillance as well as to formulate recommendations for clinical treatment, quality of life, and genetic counselling of CMMRD patients in one comprehensive guideline. With these guidelines we aim at assisting clinical management of people with CMMRD in Europe and beyond. These guidelines do not represent nor intend to be a legal standard of care, they should support clinical decision making.

## Methods

A CMMRD Guideline Group (GG) was established comprising clinicians specialised in clinical genetics, paediatric (neuro-)oncology and haematology, neuro-surgery, (paediatric) gastroenterology, radiology, pathology, and clinical psychology, with expert experience in the management of CMMRD, and (molecular) geneticists specialised in the diagnosis of CMMRD. The nineteen members of the GG were led by a Core Working Group (CWG) consisting of eight members including one patient representative.

Based on the defined scope of the guidelines, PICO (Population, Intervention, Comparison, Outcome) questions were formulated by the CWG and approved by the GG. Based on these PICO questions, Agència de Qualitat i Avaluació Sanitàries de Catalunya (AQuAS) extracted 332 articles from three databases (PubMed, Cinhal and Embase) using the following terms: CMMRD [Title/Abstract] OR CMMR-D [Title/Abstract] OR “constitutional mismatch repair deficiency” [Title/Abstract] OR “constitutive mismatch repair deficiency” [Title/Abstract] OR “biallelic mismatch repair deficiency” [Title/Abstract] OR bMMRD [Title/Abstract] OR “mismatch repair cancer syndrome” [Title/Abstract] OR “OMIM 276300”. Another 20 articles were selected using citation searching. From these 352 articles, 258 were excluded as they were conference articles, were not written in English, were not responding to any of the questions of interest, were not referring to individuals with CMMRD, and/or contained data already included from other references. The remaining 94 articles were summarised by AQuAS in a comprehensive literature review.

The CWG members drafted recommendations for CMMRD *diagnosis*, *genetic counselling*, *surveillance*, *quality of life*, and *clinical management* building on this literature review with additional articles they identified and their expert knowledge. These recommendations were approved by the GG and then subjected to a modified Delphi process. Delphi is a structured communication technique in which opinions of a large number of experts are assessed on a topic in which there is no consensus. Experts included in this exercise were all members of the CWG and the GG as well as 53 external experts identified by the CWG and the GG together. The Delphi survey consisted of two rounds, in which the threshold for consensus was defined by a simple majority of the survey participants agreeing with the recommendation ( > 60% rated ‘agree’ or ‘totally agree’). Recommendations were graded using a 4-point Likert scale (totally disagree, disagree, agree, totally agree) and a justification for the given rating was obligatory. Even if the consensus threshold was met in the first Delphi round, recommendations were still modified if a stronger consensus was thought achievable from written responses. The facilitator of the Delphi survey provided anonymised summaries of the experts’ decisions after each round as well as the reasons they provided for their judgements.

As is typical for many rare diseases, the volume of peer-reviewed evidence available to consider for these guidelines was small and came from a limited number of articles, which typically reported on individual cases or small series. To balance the weight of both published evidence and quantify the wealth of expert experience and knowledge, we have used the following scale to grade the recommendations: (i) *strong*: Expert consensus AND consistent evidence; (ii) *moderate*: Expert consensus WITH inconsistent evidence AND/OR new evidence likely to support the recommendation, and (iii) *weak*: Expert majority decision WITHOUT sufficient evidence. Expert consensus (an opinion or position reached by a group as whole) or expert majority decision (an opinion or position reached by the majority of the group) is established after reviewing the results of the modified Delphi approach within the CWG.

Recommendations were written in one of four stylistic formats: Should, Should Probably, Should Probably Not, Should Not:

- Should & Should Not, were taken to mean most well-informed people (those who have considered the evidence) would take this action.
- Should Probably & Should Probably Not, were taken to mean the majority of informed people would take this action, but a substantial minority would not.

The full details of the guideline including literature search, reference list and Delphi process can be found on the ERN GENTURIS website: https://www.genturis.eu/l=eng/Guidelines-and-pathways/Clinical-practice-guidelines.html.

## Results

Eighty-two recommendations were formulated (Table 1). After two Delphi rounds, an agreement of 68 to 100% (median 92) was reached for all recommendations and their strength was graded as weak (*n* = 5), moderate (*n* = 23) or strong (*n* = 54). The high rate of weak and moderate recommendations is mainly due to the paucity of data in the literature.

**Table 1:** Recommendations. a: Diagnosis recommendations. b: Genetic counselling recommendations. c: Surveillance recommendations. d: Quality of life recommendations. e: Clinical management recommendations.

Recommendations		Strength
**a: Diagnosis recommendations**		
	**Indications for testing**	
**Rec. 1**	CMMRD testing should be offered to all cancer patients who reach a minimum of three scoring points according to the revised C4CMMRD indication criteria (Table 2).	Strong
**Rec. 2**	CMMRD testing should be offered to all cancer patients aged <18 years with a tumour that has a paediatric-high* tumour mutational burden (TMB), regardless of presence or absence of a somatic *POLE* or *POLD1* pathogenic variant. *(Gröbner et al. [61])	Strong
**Rec. 3**	CMMRD testing should be offered to all cancer patients with a tumour that has expression loss of one or more of the four MMR proteins by immunohistochemical staining in neoplastic and in non-neoplastic cells including tumour infiltrating leukocytes and/or endothelial cells.	Strong
**Rec. 4**	CMMRD testing should be offered to all cancer patients aged <18 years in whom a heterozygous (likely) pathogenic variant in one of the MMR genes was found by germline sequencing.	Strong
**Rec. 5**	A family history assessment and physical examination should be performed for any patient who fulfils inclusion criteria of CMMRD testing as described in Rec. 2–4.	Strong
**Rec. 6**	CMMRD testing should probably be offered following an interdisciplinary discussion to all children suspected to have sporadic NF1/Legius syndrome without cancer and without an *NF1*/*SPRED1* germline (L)PV after comprehensive genetic analysis and who have at least one additional feature defined by the C4CMMRD guidelines (Suerink et al. [20], Table 3).	Strong
	**Testing strategy**	
**Rec. 7**	Any testing strategy should aim to come to a definite diagnosis that either confirms or refutes CMMRD in the patient, and to identify the causative variants in the relevant MMR gene.	Strong
**Rec. 8**	Wherever possible, CMMRD testing of a patient with a (pre-)malignancy should include immunohistochemical staining of all four MMR proteins in tumour tissue to determine MMR protein expression in neoplastic and in non-neoplastic cells, including tumour infiltrating leukocytes and/or endothelial cells.	Strong
**Rec. 9**	The laboratory performing genetic CMMRD testing should be able to offer transcript analysis of all four MMR genes and should be able to apply assays that circumvent potential diagnostic pitfalls that result from the high homology of *PMS2* and its pseudogene *PMS2CL* (either by partnership with a different laboratory or in their own laboratory).	Strong
**Rec. 10**	The laboratory performing genetic CMMRD testing of an index patient with a (pre-)malignancy should probably have one or more validated ancillary assay(s) available (either by partnership with a different laboratory or in their own laboratory) that can definitively confirm or refute the diagnosis of CMMRD if genetic testing renders an inconclusive result (the currently available ancillary assays testing for constitutional MMR deficiency are listed in Table 4).	Strong
**Rec. 11**	The laboratory performing genetic CMMRD testing of an index patient without a (pre-)malignancy should have one or more validated ancillary assay(s) available (either by partnership with a different laboratory or in their own laboratory) that can definitively confirm or refute the diagnosis of CMMRD if genetic testing renders an inconclusive result (the currently available ancillary assays testing for constitutional MMR deficiency are listed in Table 4).	Strong
	**Diagnostic criteria**	
**Rec. 12**	The diagnosis of CMMRD should be considered confirmed in an individual fulfilling one or more of the suggested criteria for CMMRD testing (Rec.1, Rec.2, Rec.3, Rec.4, Rec.6) if, according to the Table “Criteria for the confirmation of CMMRD” (Table 5): (i) in one of the four MMR genes, two variants classified according to internationally accepted classification criteria* as (likely) pathogenic (PV or LPV) are identified and are confirmed to be located in trans (note that in some cases additional criteria need to be fulfilled); OR (ii) in one of the four MMR genes, one of two variants identified and confirmed to be located in trans is classified as a PV or LPV or variant of unknown significance (VUS) and the other one is classified as a VUS and one or more clinically validated ancillary test results is consistent with a CMMRD diagnosis; OR (iii) in one of the four MMR genes, one variant is identified and classified as a PV or LPV or VUS and there is evidence for (a) faulty splicing not explained by the identified variant or (b) reduced expression of the wild-type allele by transcript analysis and one or more clinically validated ancillary test results is consistent with a CMMRD diagnosis; OR (iv) no MMR gene variant classified as a PV or LPV or VUS is identified, but one or more clinically validated ancillary test results is consistent with a CMMRD diagnosis and there is evidence by transcript analysis for (a) faulty splicing or (b) reduced expression of the wild-type allele(s) of one of the MMR genes. *ClinGen InSiGHT Hereditary Colorectal Cancer/Polyposis Variant Curation Expert Panel Specifications to the ACMG/AMP Variant Interpretation Guidelines for MMR genes.	Moderate
**Rec. 13**	Cancer patients fulfilling the suggested criteria for CMMRD testing, Rec.1, Rec.2 or Rec.4, in whom the diagnosis CMMRD cannot be confirmed, should probably be tested for a germline (likely) pathogenic variant in the exonuclease domains of *POLE* and *POLD1*.	Strong
**Rec. 14**	In a deceased cancer patient fulfilling one or more of the suggested criteria for CMMRD testing (Rec.1, Rec.2, Rec.4) for whom no germline DNA/RNA is available and the diagnosis of CMMRD cannot be confirmed by one or more of the criteria outlined in Rec.12 and Table 5, the diagnosis of CMMRD should be considered confirmed if immunohistochemical staining shows expression loss of one or more MMR proteins in neoplastic and in non-neoplastic cells, including tumour infiltrating leukocytes and/or endothelial cells, of the patient and expression in an appropriate positive control.	Moderate
**b: Genetic counselling recommendations**		
**Rec. 1**	Genetic counselling should be offered to parents and siblings of a confirmed CMMRD patient, preferentially by a multidisciplinary team with knowledge of CMMRD, consisting of a medical geneticist, a paediatric oncologist and a psychologist.	Strong
**Rec. 2**	To confirm their carrier status, parents of a CMMRD patient should be offered genetic testing for the (likely) pathogenic MMR gene variants found in their child.	Strong
**Rec. 3**	Cascade genetic testing for (likely) pathogenic variants should be offered to all adult relatives of a CMMRD patient, in both parental branches.	Strong
**Rec. 4**	Siblings of a genetically confirmed CMMRD patient should be offered genetic CMMRD testing regardless of age and phenotype.	Strong
**Rec. 5**	When performing CMMRD predictive testing in a minor or prenatal testing, pros and cons of revealing results of genetic testing regarding Lynch syndrome should be discussed on a case-by-case basis with the parents and the patient depending on their age.	Moderate
**Rec. 6**	If the diagnosis of CMMRD is not confirmed by the identification of two (likely) pathogenic variants in one MMR gene but by ancillary tests in the patient, siblings should probably be offered ancillary tests to exclude a CMMRD diagnosis for them.	Moderate
**Rec. 7**	Prenatal or preimplantation genetic testing should be discussed with parents of reproductive age of a CMMRD patient.	Strong
**Rec. 8**	Prenatal or preimplantation genetic testing should be discussed with couples of reproductive age if both carry a pathogenic variant in the same MMR gene.	Strong
**Rec. 9**	Testing the partner of a CMMRD patient for the MMR gene involved should probably be discussed during genetic counselling, considering possible consanguinity, common founder effect, and family history suggestive of Lynch syndrome.	Strong
**Rec. 10**	The partner of a Lynch syndrome carrier should be offered genetic testing of MMR genes if consanguinity is reported by the couple or the partner is coming from a population with a known founder variant or the family history of the partner is suggestive of Lynch syndrome and genetic testing has not been performed yet.	Strong
**Rec. 11**	The partner of a Lynch syndrome carrier should not be actively offered genetic testing of MMR genes in the absence of consanguinity, a known founder mutation or a family history suggestive of Lynch syndrome.	Moderate
**Rec. 12**	The child of a Lynch syndrome carrier should probably be offered CMMRD testing, if the child has clinical features that add up to ≥ 2 C4CMMRD scoring points according to the revised criteria (Table 2: scoring points assigned to additional features in the patient).	Strong
**c: Surveillance recommendations**		
**Rec. 1**	CMMRD patients and/or their parents should be educated about tumour risks associated with CMMRD.	Strong
**Rec. 2**	CMMRD patients and/or their parents should be educated about symptoms related to the main tumours, especially dyspnoea and superior vena cava syndrome for mediastinal lymphomas, symptoms associated with pancytopenia for leukaemia, neurological symptoms for brain tumours, and bleeding for colorectal tumours.	Strong
**Rec. 3**	Pros and cons should be discussed among the CMMRD patient and/or their parents and clinician to make a joint decision to participate in a surveillance program.	Strong
**Rec.4**	CMMRD patients and/or their parents should probably be encouraged to communicate their screening results in research projects or databases to improve knowledge on CMMRD.	Strong
**Rec. 5**	In children and adults with CMMRD, clinical examination should be performed every 6 months.	Strong
**Rec. 6**	Brain MRI should probably start at the initial CMMRD diagnosis or at least at the age of 2 years.	Strong
**Rec. 7**	In CMMRD patients up to age 20 years, brain MRI should be performed every 6 months.	Strong
**Rec. 8**	In CMMRD patients older than 20 years, a brain MRI should be performed at least annually.	Moderate
**Rec. 9**	The first brain MRI should probably be performed with contrast enhancement for all CMMRD patients.	Moderate
**Rec. 10**	In patients with CMMRD without a previous brain tumour, MRI should probably include anatomical sequence T2 FLAIR (if possible in 3D) combined with MRI diffusion sequence.	Moderate
**Rec.11**	In patients with CMMRD with a previous brain tumour, MRI should include anatomical sequences T2-FLAIR, diffusion sequence, and T1+ contrast enhancement if possible in 3D.	Moderate
**Rec. 12**	Abdominal ultrasound should probably not be performed to screen for abdominal lymphomas in CMMRD patients.	Weak
**Rec. 13**	Blood counts should probably not be performed to screen for haematological (pre-)malignancies in CMMRD patients.	Weak
**Rec. 14**	Colonoscopy should be performed at least annually in CMMRD patients and should probably start from the age of 6 years in children with CMMRD.	Strong
**Rec. 15**	Upper gastrointestinal endoscopy should be performed annually in CMMRD patients and should probably start at the same age as colonoscopy or at least at the age of 10 years.	Weak
**Rec. 16**	Upper endoscopy should probably visualize the whole duodenum and include careful inspection of the ampullary region in CMMRD patients.	Weak
**Rec. 17**	Upper endoscopy and colonoscopy should probably be done with coloration in the context of CMMRD.	Weak
**Rec. 18**	The frequency of upper or lower endoscopy should probably increase up to 6 months-interval once polyps are detected in the context of CMMRD.	Strong
**Rec. 19**	Digestive tract surveillance for CMMRD patients, including children, should probably be done in a centre with gastroenterologists experienced in Lynch syndrome screening.	Moderate
**Rec. 20**	The interval between two digestive tract examinations should not exceed 12 months for CMMRD patients.	Strong
**Rec. 21**	Video capsule endoscopy should be performed annually in CMMRD patients and should probably be performed from the age of 10 years.	Moderate
**Rec. 22**	Gynaecologic surveillance should probably be performed annually from age 20 years in CMMRD patients and should include clinical examination and transvaginal ultrasound.	Strong
**Rec. 23**	Prophylactic hysterectomy should probably be discussed once family planning of the CMMRD patient is completed.	Moderate
**Rec. 24**	Annual urine cytology and urine dipstick should probably not be offered to CMMRD patients.	Moderate
**Rec. 25**	Abdominopelvic ultrasound for gynaecological and urinary tract cancer screening should probably be offered annually to CMMRD patients, starting at 20 years of age.	Strong
**Rec. 26**	Breast cancer screening should probably follow general population guidelines for CMMRD patients.	Moderate
**Rec. 27**	Whole body MRI should probably be offered to CMMRD patients at least once, at diagnosis or when anaesthesia is no longer required, for a general screening of low-grade tumours and malformations to guide targeted screening.	Strong
**Rec. 28**	Resection or specific surveillance of low-grade lesions should be offered to CMMRD patients.	Strong
**Rec. 29**	Even though evidence of its efficacy in screening is still weak in CMMRD, whole-body MRI should probably be discussed with CMMRD patients as an option for annual surveillance.	Moderate
**d: Quality of life recommendations**		
**Rec. 1**	Psychological support should be offered to the patient and the family during the entire process of evaluation before the diagnosis of CMMRD.	Strong
**Rec. 2**	Psychological support should be offered to patients with CMMRD and their families at any time during treatment and cancer surveillance.	Strong
**Rec. 3**	Age adapted education about CMMRD should probably be offered to CMMRD patients and their families.	Strong
**Rec. 4**	Healthcare professionals involved in diagnosis and surveillance should address the psychosocial implications of a diagnosis of CMMRD.	Strong
**e: Clinical management recommendations**		
**Rec. 1**	Multiple patients with CMMRD have been cured from a cancer diagnosis. Thus, in a CMMRD patient diagnosed with cancer, a curative approach should be considered and evaluated.	Strong
**Rec. 2**	For several cancer types, no CMMRD specific treatment recommendations exist. Treatment of patients with CMMRD related neoplasms should, therefore, probably be discussed in a multidisciplinary board with a treating physician, an expert for the patient’s cancer type as well as a CMMRD expert.	Strong
**Rec. 3**	Patients with CMMRD associated neoplasms should probably be included in clinical trials whenever possible.	Strong
**Rec. 4**	CMMRD is probably not a contraindication for radiotherapy, if indicated.	Moderate
**Rec. 5**	CMMRD is probably not a contraindication for haematopoietic stem cell transplantation, if indicated.	Moderate
**Rec. 6**	Temozolomide should probably be avoided in patients with CMMRD-associated high-grade glioma.	Strong
**Rec. 7**	The use of immunotherapy with a PD1 inhibitor should be considered for CMMRD patients with high-grade glioma, preferentially within a clinical trial.	Strong
**Rec. 8**	CMMRD-associated low grade glioma should probably be resected whenever possible without excessive neurological risks.	Strong
**Rec. 9**	Front-line treatment of CMMRD-associated medulloblastoma should probably not differ from treatment of sporadic medulloblastoma/primitive neuro-ectodermal tumours.	Moderate
**Rec. 10**	In case of CMMRD-associated non-Hodgkin lymphoma, chemotherapy should probably be similar to the treatment of the same tumour without CMMRD.	Moderate
**Rec. 11**	In case of a second primary non-Hodgkin lymphoma in a CMMRD patient, standard first-line treatment adapted to the non-Hodgkin lymphoma subtype taking into account cumulative doses of chemotherapy previously received should probably be given rather than a relapse treatment.	Moderate
**Rec. 12**	In case of CMMRD-associated leukaemia, chemotherapy should probably be similar to the treatment of the same cancer without CMMRD.	Moderate
**Rec. 13**	In case of diagnosis of a cancer of the Lynch spectrum in a CMMRD patient, treatment guidelines designed for patients with Lynch syndrome associated tumours should be followed.	Strong
**Rec. 14**	Immunotherapy should be recommended as front-line treatment of large, unresectable or metastatic colorectal tumours in a CMMRD patient	Strong
**Rec. 15**	Immunotherapy should be performed front-line for all extra-colorectal Lynch-related tumours in CMMRD patients ideally in therapeutic trials.	Strong
**Rec. 16**	Immunotherapy should be encouraged in interdisciplinary discussions for any non-Lynch related tumour at any time during treatment (diagnosis or relapse) of a CMMRD patient, especially if standard therapeutic guidelines offer only low chance of cure.	Moderate
**Rec. 17**	CMMRD patients with multiple colonic adenomas should probably be surgically managed in line with general practice for other polyposis syndromes.	Strong
**Rec. 18**	CMMRD patients may present with multiple tumours at the same time or may develop additional tumours during treatment. Thus, cancer surveillance around the time of diagnosis and during the period of cancer treatment should be offered.	Strong
**Rec. 19**	In CMMRD patients with a suspected relapse, a second primary disease should be considered. This may influence the treatment choice.	Strong
**Rec. 20**	In case of relapse of a CMMRD-associated tumour, molecular analysis of samples at initial diagnosis and relapse should be performed to differentiate a relapse from a second primary tumour.	Strong
**Rec. 21**	Fresh tumour specimens should be collected and stored (or directly molecularly analysed) whenever possible and if the CMMRD patient and/or their family approves. This may be relevant for research as well as for clinical purposes (e.g. see Rec 19).	Strong
**Rec. 22**	Advantages and potential side effects of preventive treatment with acetylsalicylic acid should probably be discussed with CMMRD patients.	Moderate
**Rec. 23**	CMMRD patients with IgG/A reduced levels/deficiency should not be treated to compensate for the inherent deficit in the absence of clinical manifestations.	Strong

**Fig. 1:**
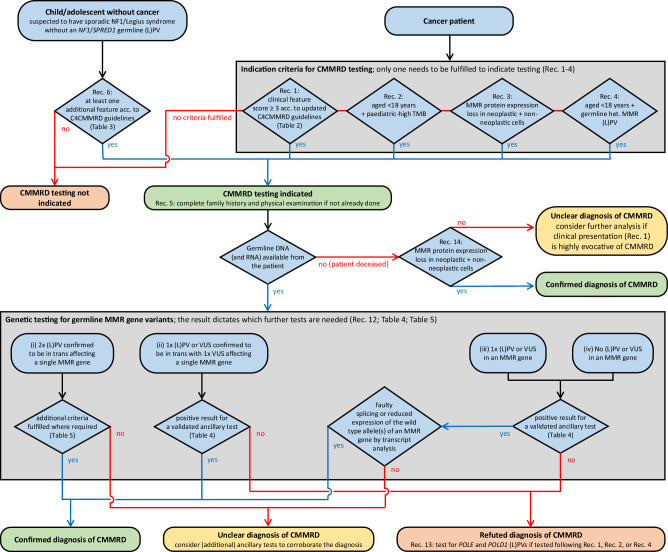
Diagnostic decision tree. Rectangles with rounded corners indicate clinical, genetic, and diagnostic status of the patient prior to and during diagnostic work-up (blue filling) as well as after diagnostic work-up (green, yellow and red filling). Diamonds with blue filling indicate decision points that can either be fulfilled (yes, follow the blue arrow) or not be fulfilled (no, follow the red arrow).

Fourteen recommendations for the *diagnosis* of CMMRD (Table 1a) provide indication criteria for CMMRD testing based on (revised) C4CMMRD indication criteria for CMMRD testing in paediatric/young adult cancer patients (Table 2) and in children suspected of having sporadic NF1/Legius syndrome without a cancer in whom genetic testing cannot confirm either of these suspected diagnoses (Table 3). These recommendations also include criteria based on the phenotype of the tumour in paediatric cancer patients (Table 1a). Further recommendations define CMMRD testing strategies, which may include ancillary tests (Table 4), and criteria for a CMMRD diagnosis (Table 5 and Fig. 1).

**Table 2:** Revised C4CMMRD indication criteria for CMMRD testing in cancer patients^a^.

CMMRD testing is indicated in a cancer patient reaching ≥ 3 points.	
C4CMMRD scoring points assigned to (pre-)malignancies in the patient (at least one point is mandatory):	
Carcinoma of the Lynch syndrome (LS) spectrum^b^ and/or a high-grade dysplastic adenoma of the digestive tract at age < 25 years	3 points
Multiple colorectal adenomas at age < 25 years and no genetic diagnosis/explanation upon testing for polyposis syndromes	3 points
T-cell lymphoblastic lymphoma (T-LBL) at age < 18 years	2 points
WHO grade III or IV glioma at age < 25 years	2 points
Any other malignancy at age < 18 years	1 point
**C4CMMRD scoring points assigned to additional features in the patient (optional):**	
Clinical sign of Neurofibromatosis type 1 (NF1)^c^ and/or ≥***4*** hyperpigmented and/or hypopigmented skin alterations with Ø^d^ >1 cm	2 points
2 or 3 hyperpigmented and/or hypopigmented skin alterations with Ø >1 cm (Do not count if two points are already given for “Clinical sign of NF1 and/or ≥ 4 hyperpigmented and/or hypopigmented skin alterations with Ø >1 cm”)	1 point
Multiple pilomatrixomas	2 points
One pilomatrixoma	1 point
Agenesis of the corpus callosum	1 point
Non-therapy-induced cavernoma	1 point
Multiple developmental venous anomalies (DVAs, also known as cerebral venous angiomas) in separate regions of the brain	2 points
Paediatric systemic lupus erythematosus	1 point
Deficiency/reduced levels of IgG2/4 and/or IgA	1 point
**C4CMMRD scoring points assigned to additional features in the family (optional):**	
Consanguineous parents	1 point
Diagnosis of LS in a first-degree or second-degree relative	2 points
Carcinoma from LS spectrum^b^ before the age of 60 years in a first-degree, second-degree, and/or third-degree relative	1 point
A sibling with a (pre-)malignancy assigned two or three C4CMMRD scoring points	2 points
A sibling with any type of childhood malignancy	1 point

*C4CMMRD* Care for CMMRD, *(L)PV(s)* (likely) pathogenic variant(s), *WHO* World Health Organization, *NF1* Neurofibromatosis type 1.

^a^Original C4CMMRD criteria: Wimmer et al. Diagnostic criteria for constitutional mismatch repair deficiency syndrome: suggestions of the European consortium ‘care for CMMRD’ (C4CMMRD). J Med Genet 2014; 51(6):355-65.

^b^Colorectal, endometrial, small bowel, urothelial, gastric, ovarian, and biliary tract cancer.

^c^Clinical sign in the patient used for the diagnosis of NF1 according to: Legius et al. Revised diagnostic criteria for neurofibromatosis type 1 and Legius syndrome: an international consensus recommendation. Genet Med 2021; 23(8):1506–1513.

^d^Diameter.

**Table 3:** Selection strategy for CMMRD counselling and testing in a child suspected to have NF1/Legius syndrome (without cancer) and a negative outcome of *NF1*/*SPRED1* germline mutation analysis.

Prerequisites:
► Suspicion of NF1 due to the presence of at least one diagnostic NF1 feature^a^, including at least two hyperpigmented skin patches reminiscent of CALMs.
► No (likely) pathogenic germline variant in *NF1* and *SPRED1* detected using comprehensive and highly sensitive mutation analysis protocols^b^.
► Absence of diagnostic NF1 sign(s) in both parents.
**Additional features, at least one (either in the family or in the patient) is required:**
In the family:
► Consanguineous parents.
► Genetic diagnosis of Lynch syndrome in one or both parental families.
► Sibling with diagnostic NF1 sign(s).
► A (deceased) sibling^c^ with any type of childhood malignancy.
► One of the following carcinomas of the Lynch syndrome spectrum: Colorectal, endometrial, small bowel, urothelial, gastric, ovarian, and biliary tract cancer, before the age of 60 years in a first-degree or second-degree relative.
In the patient:
► Atypical CALMs (irregular borders and/or pigmentation).
► Multiple hypopigmented skin patches.
► One or more pilomatrixoma(s) in the patient.
► Agenesis of the corpus callosum.
► Non-therapy-induced cavernoma.
► Multiple developmental venous anomalies (DVA, also known as cerebral venous angiomas) in separate regions of the brain.

*CMMRD* Constitutional mismatch repair deficiency, *NF1* Neurofibromatosis type 1, *CALMs* Café-au-lait macules.

^a^Legius et al. Revised diagnostic criteria for neurofibromatosis type 1 and Legius syndrome: an international consensus recommendation. Genet Med 2021; 23(8):1506–1513.

^b^Analysis protocol should include methods that identify and/or characterise unusual splice variants.

^c^This can be expanded to second-degree and third-degree relatives in populations with a high prevalence of founder mutations.

**Table 4:** Ancillary tests for assessing constitutional MMR deficiency.

Validated test^#^	CMMRD confirmed	CMMRD refuted
Germline Microsatellite instability (gMSI) testing acc. to Ingham et al. [28].^a^	gMSI ratios of at least two (usually all three) microsatellite markers are above the validated laboratory’s internal thresholds	Not possible by the test
Constitutional MSI (cMSI) testing acc. to Gallon et al. [25, 26].^b^	cMSI score above the validated laboratory’s internal thresholds	cMSI score within the score range of negative controls
High-sensitivity MSI (hsMSI) testing acc. to González-Acosta et al. [27].^c^	hsMSI score above the validated laboratory’s internal thresholds	hsMSI score within the score range of negative controls
Ex vivo MSI (evMSI) + methylation tolerance acc.to Bodo et al. [22].^d^	evMSI and methylation tolerance above the validated laboratory’s internal thresholds	evMSI and methylation tolerance within the range of negative controls
MMRDness testing by low-pass whole-genome sequencing/ LOGIC assay in blood leukocytes acc. to Chung et al. [24].^e^	MMRDness score above the validated laboratory’s internal thresholds	MMRDness score within the score range of negative controls

*MMR* Mismatch repair, *CMMRD* Constitutional mismatch repair deficiency, *MSI* microsatellite instability, *acc.* according, *PV* pathogenic variant, *(L)PV* (likely) pathogenic variant.

^#^Validation cohort should include (i) at least eight CMMRD patients with different genotypes with respect to PVs and affected gene (for each of the four MMR genes at least one patient should be included), (ii) a large number of negative controls consisting of at least twenty adult individuals aged > 40 years without cancer history and without a MMR gene (L)PV, (iii) at least ten confirmed MMR gene PV heterozygotes and, if available, (iv) POLE and POLD1 PV heterozygotes.

^a^Ingham et al. Simple detection of germline microsatellite instability for diagnosis of constitutional mismatch repair cancer syndrome. Hum Mutat 2013; 34:847–52.

^b^Gallon et al. A sensitive and scalable microsatellite instability assay to diagnose constitutional mismatch repair deficiency by sequencing of peripheral blood leukocytes. Hum Mutat 2019; 40(5):649–655.

^b^Gallon et al. Constitutional microsatellite instability, genotype, and phenotype correlations in Constitutional Mismatch Repair Deficiency. Gastroenterology 2023; 164(4):579–592.

^c^González-Acosta et al. High-sensitivity microsatellite instability assessment for the detection of mismatch repair defects in normal tissue of biallelic germline mismatch repair mutation carriers. J Med Genet 2020; 57(4):269–273.

^c^Marín et al. A Validated Highly Sensitive Microsatellite Instability Assay Accurately Identifies Individuals Harboring Biallelic Germline PMS2 Pathogenic Variants in Constitutional Mismatch Repair Deficiency. Clin Chem 2024; 70(5):737–746.

^d^Bodo et al. Diagnosis of Constitutional Mismatch Repair-Deficiency Syndrome Based on Microsatellite instability and Lymphocyte Tolerance to Methylating Agents. Gastroenterology 2015; 149:1017–29.

^e^Chung et al. Genomic Microsatellite Signatures Identify Germline Mismatch Repair Deficiency and Risk of Cancer Onset. J Clin Oncol 2023; 41(4):766–777.

**Table 5:** Criteria for the confirmation of CMMRD.

Genotype	MMR gene genetic testing reason					
Germline MMR gene variants identified (if two variants are identified, they must be confirmed to be in trans)	C4CMMRD criteria for cancer patient fulfilled (Rec. 1)	Cancer < 18 years with paediatric high TMB (Rec. 2)	Cancer with MMR protein expression loss in neoplastic and non-neoplastic cells including tumour infiltrating lymphocytes and/or endothelial cells (Rec. 3)	Cancer < 18 years with heterozygous germline MMR gene (L)PV (Rec. 4)	C4CMMRD criteria for children without cancer suspected to have NF1/Legius syndrome and a negative *NF1*/*SPRED1* mutation analysis (Rec. 6)	Incidental finding in WES or WGS performed for other reasons in a patient without cancer
PV/PV	√	√	√	√	√	√
PV/LPV	√	√(PPAP-)	√	√	√	√(AT + )
LPV/LPV	√	√(PPAP-)	√(AT + )	√(AT + )	√(AT + )	√(AT + )
(L)PV/VUS	√(AT + )	√(AT + )	√(AT + )	√(AT + )	√(AT + )	√(AT + )
VUS/VUS	√(AT + )	√(AT + )	√(AT + )	NA	√(AT + )	NA
(L)PV/X	√(AT + ; mRNA + )	√(AT + ; mRNA + )	√(AT + ; mRNA + )	√(AT + ; mRNA + )	√(AT + ; mRNA + )	NA
VUS/X	√(AT + ; mRNA + )	√(AT + ; mRNA + )	√(AT + ; mRNA + )	NA	√(AT + ; mRNA + )	NA
X/X	√(AT + ; mRNA + )	√(AT + ; mRNA + )	√(AT + ; mRNA + )	NA	√(AT + ; mRNA + )	NA

*C4CMMRD* Care for CMMRD, *MMR* mismatch repair, *TMB* Tumour mutation burden, *PV* Pathogenic variant, *LPV* Likely pathogenic variant, *(L)PV* Likely pathogenic or pathogenic variant, *VUS* variant of unknown significance, *WES* Whole exome sequencing, *WGS* Whole genome sequencing; alleles are separated by / and X indicates one allele without an identifiable (L)PV or VUS.

*NA* not applicable.

√ = CMMRD confirmed without further ancillary test or transcript analysis.

√(PPAP-) = CMMRD confirmed without further ancillary test if *POLE*/*POLD1* germline mutation excluded (i.e., polymerase proofreading associated polyposis negative: PPAP-).

√(AT + ) = CMMRD confirmed if validated ancillary test positive for CMMRD (AT + ).

√(AT + ; mRNA + ) = CMMRD confirmed if validated ancillary test positive for CMMRD (AT + ) and evidence by transcript analysis for (a) faulty splicing (not explained by the identified variant) or (b) reduced expression of the wild-type allele(s) (mRNA + ).

Twelve recommendations for *genetic counselling* (Table 1b) include recommendations for predictive testing in relatives of CMMRD patients, for prenatal and preimplantation CMMRD testing and for MMR gene analysis in partners of CMMRD and LS patients.

Twenty-nine recommendations for *surveillance* are presented in Table 1c and summarised in Table 6. They include six general recommendations (recommendations (Rec.) 1–5 and 28), six specific recommendations for brain tumour surveillance by MRI, two for haematological malignancy surveillance, eight for gastro-intestinal surveillance, and five for surveillance in adulthood for other LS-related tumours, such as gynaecological or urinary tract tumours, and breast cancer. Additionally, two recommendations address the place of whole body MRI in CMMRD surveillance. All recommendations contain information on the starting age, the frequency, and modalities of the surveillance.

**Table 6:** Summary of surveillance recommendations.

Exam		Frequency	Period	Evidence^a^
Clinical examination		Every 6 months	From diagnosis	Strong
Brain MRI		Every 6 months	2–20 years	Strong
		Annually	From 20 years	Moderate
Colonoscopy		Annually^b^	From 6 years	Strong
Upper gastrointestinal endoscopy		Annually^b^	Simultaneously with colonoscopy or at least from age 10 years	Weak
Video capsule endoscopy		Annually	From 10 years	Moderate
Gynaecologic	Surveillance (clinical examination & transvaginal ultrasound)	Annually	From 20 years	Strong
	Prophylactic surgery	Not applicable	Discuss once family planning is completed	Moderate
Abdominopelvic ultrasound for gynaecological and urinary tract cancer screening		Annually	From 20 years	Strong
Whole body MRI		At least once	At diagnosis or when anaesthesia is no longer required	Strong
		Discuss optional annual imaging		Moderate

^a^This grading is based on published articles and expert consensus.

^b^Interval should be increased to once every 6 months once polyps are detected.

Four recommendations for *quality of life* mainly address psychological support and age adapted patient and family education (Table 1d).

Twenty-three recommendations for clinical management consist of general recommendations on malignancy treatment (including radiation therapy and stem cell transplantation; Table 1e). Specific recommendations are given for ICI therapy of high-grade glioma, colorectal cancer, other LS-related and non-LS related malignancies and for chemotherapy of non-Hodgkin lymphoma and leukaemia. Management of polyposis, low-grade glioma, medulloblastoma, suspected tumour relapse and IgG/A production deficits, surveillance during tumour treatment, and colorectal cancer prevention with acetylsalicylic acid are also covered.

## Discussion

These are the first comprehensive guidelines addressing the most important aspects of care for CMMRD across five sections: *diagnosis*, *genetic counselling*, *surveillance*, *quality of life*, and *clinical management*. Previous guidelines focused only on one or two of these topics, mainly diagnosis and surveillance, and mentioned other aspects of CMMRD care, such as clinical management, genetic counselling, and quality of life, only in their discussions. In the following, evidence for the recommendations given here and their comparison with recommendations in existing guidelines for the diagnosis and surveillance of CMMRD are provided.

### Diagnosis

#### Indications for testing

The recommendations 1–4 and 6 address the question of which children/young adults should be tested for CMMRD. The recommendations 1 and 6 of these guidelines are based on the C4CMMRD guidelines for the clinical indication for CMMRD testing in cancer patients ([15], Table 2) and in children suspected to have sporadic NF1/Legius syndrome without cancer and without an *NF1*/*SPRED1* germline PV after comprehensive genetic analysis ([20], Table 3). While the latter guidelines are included here without any change (Table 3), the C4CMMRD guidelines for the clinical indication for CMMRD testing in cancer patients, a 3-point scoring system (Table 2), have been adapted to include new knowledge. Specifically, two tumour types should now be differently named. CMMRD-associated non-Hodgkin lymphoma (NHL) of the T-cell lineage falls mainly in the entity of T-cell lymphoblastic lymphoma. Supra-tentorial primitive neuroectodermal tumours no longer exist as a specific tumour type and CMMRD-associated brain tumours previously classified as such fall mainly into the group of diffuse high-grade gliomas. Furthermore, multiple developmental venous anomalies (also known as cerebral venous angiomas) of the brain, which are frequent in CMMRD (73–100% patients) [16, 40, 41] were included as a new feature with two points as DVA are rare (1.6%) in the general paediatric population. Note however, that a single DVA was found in 10% of NF1 patients analysed, most of whom had either low-grade or high-grade gliomas, in 14% of patients with Lynch syndrome and high-grade gliomas, and in 6% of all sporadic patients with high-grade gliomas, while multiple DVA were found in 83% of CMMRD with high-grade gliomas and 3% of the analysed NF1 patients, but in none of the sporadic or Lynch syndrome patients with HGGs [17]. Paediatric systemic lupus erythematosus, which is rare in the general population (prevalence <1/25,000 children), was found in a total of six of more than 200 reported CMMRD patients and is, hence, also significantly overrepresented in CMMRD patients [42, 43]. This feature is newly included with one point. So far, unpublished results of the Gustave Roussy university hospital, which systematically applied the original C4CMMRD criteria, showed that assigning two points to two or more hyperpigmented and/or hypopigmented skin alterations was too inclusive and led to CMMRD testing in many non-CMMRD patients causing often unnecessary anxiety in patients and their families as well as extensive diagnostic effort to refute the diagnosis. This feature was assigned only one point in the revised criteria. However, clinical signs of NF1 and/or ≥4 hyperpigmented and/or hypopigmented skin alterations with a diameter over 1 cm remained weighted with two points. Note that it has been shown in a small number of patients with CMMRD that an NF1 phenotype may result from a (postzygotic) *NF1* PV identifiable in blood leukocytes [44, 45]. Therefore, it is advisable to consider CMMRD also in genetically confirmed (mosaic) NF1 patients who have a malignancy that is not typical of NF1, such as a paediatric diffuse high-grade glioma, a T-cell lymphoblastic lymphoma or other.

The original C4CMMRD guidelines [15] are widely used and were included also as a diagnostic entry point in recommendations from the international consensus working group on diagnostic criteria for CMMRD, consisting of members of the IRRDC and C4CMMRD [39] and the Pediatric Cancer Working Group of the AACR [29]. A tumour with a high TMB (Rec. 2) or expression loss of one or more of the four MMR proteins in neoplastic and in non-neoplastic cells (Rec. 3) are criteria for CMMRD testing recommended by the US Multi-Society Task Force on Colorectal Cancer in addition to clinical features covered by the C4CMMRD guidelines [11]. Recommendation 4 accommodates for the identification of a heterozygous (likely) pathogenic variant in one of the MMR genes in a cancer patient aged < 18 years by germline whole exome or whole genome sequencing, which is performed with increasing frequency in paediatric cancer patients [46]. Given that a second PV in the notoriously difficult to analyse *PMS2* [47], but also in the other MMR genes, may escape detection by whole exome or whole genome sequencing, such a result should entail CMMRD diagnostic work-up.

#### Testing strategy and diagnostic criteria

Some aspects of the optimal CMMRD testing strategy (Rec. 7–11) and criteria for a definitive CMMRD diagnosis (recommendations 12–14) have been discussed in previous guidelines [15, 20] and/or formulated into the diagnostic criteria provided in the international consensus working group recommendations [39]. There are several differences in the present recommendations compared to these earlier recommendations. Here, Table 4 lists ancillary tests that can confirm or refute CMMRD if genetic testing is inconclusive. In contrast to the previous recommendations [39], immunohistochemistry of the four MMR proteins is not included as an ancillary test in this list, as this approach can generate both false positive and false negative results and, therefore, is not suitable to confirm or refute CMMRD. An exception is a deceased cancer patient fulfilling one or more criteria for CMMRD testing and for whom no germline DNA is available (Rec. 14). Nonetheless, immunohistochemistry staining of all four MMR proteins in tumour tissue to determine MMR protein expression in neoplastic and in non-neoplastic cells, including tumour infiltrating leukocytes and/or endothelial cells, is recommended wherever possible to gain additional information that can strengthen the evidence for or against a CMMRD diagnosis (Rec. 8). All ancillary assays listed in Table 4 have been evaluated in large cohorts of positive and negative controls. With the exception of the gMSI assay [28], which is insensitive to MSH6-associated CMMRD, all have achieved 100% specificity and sensitivity [22, 24, 26, 27]. A positive result from any of these single ancillary tests (including the gMSI assay) can confirm a CMMRD diagnosis when genetic testing is inconclusive, provided that the assay has been thoroughly evaluated in the laboratory. This is independent of whether a malignancy defined by the international consensus working group [39] as a “CMMRD hallmark cancer” is present or not. Here, it is recommended that in patients in whom only one or no MMR variant classified as (L)PV or VUS has been identified, transcript analysis should show either faulty splicing or reduced expression of the wild-type MMR allele(s) in addition to a positive ancillary test result for a CMMRD diagnosis to be made (Rec. 12, Table 5, Fig. 1). In contrast to the international consensus working group recommendations [39], the present criteria make no distinction between a definite and a likely diagnosis. They stress that any testing strategy should aim to come to a definite diagnosis that either confirms or refutes CMMRD in the patient and to identify the causative MMR gene variants (Rec. 7). Therefore, the laboratory performing genetic CMMRD testing should be able to: (i) offer transcript analysis of all four MMR genes, (ii) apply assays that circumvent potential diagnostic pitfalls that result from the high homology of *PMS2* and its pseudogene *PMS2CL* and (iii) use one or more validated ancillary assay(s) available either in house or by partnership with a different laboratory (Rec. 9–11). Cancer patients in whom the suspected diagnosis of CMMRD cannot be confirmed should probably be tested for germline *POLE* and *POLD1* exonuclease domain variants (Rec. 13), since specific heterozygous *POLE* germline PV as well as a digenic combination of a heterozygous germline *POLE* or *POLD1* PV and a heterozygous germline MMR gene PV have been shown to cause a phenotype reminiscent of CMMRD [48, 49].

### Genetic counselling

There is discussion of whether LS carriers of reproductive age should be informed about the risk of CMMRD syndrome for their offspring and of the possibility of testing their partner before pregnancy. This is particularly relevant for the *PMS2* gene since, based on data from first degree relatives in the Colon Cancer Family Registry, the frequency of *PMS2* PV carriers in the general population is 1 in 714 and they may not present as LS due to the low penetrance of *PMS2* PV in the heterozygous state [21]. Therefore, the a priori risk for a *PMS2-*associated LS carrier to give birth to a child affected with CMMRD is estimated to be 1/2856 (1/4 × 1/714) and for a *PMS2*-associated CMMRD patient it is estimated to be 1/1428 (1/2 × 1/714). These risks are higher in cases of consanguinity of the couple or in populations with founder effects. Currently, there are no published recommendations regarding whether to test the partner of a Lynch or CMMRD syndrome patient outside the context of consanguinity or founder effect. This question was discussed at the C4CMMRD Consortium meeting in November 2022 in Paris without reaching a consensus. Indeed, such a recommendation depends on the possibilities of access to genetic testing in each country (costs, prescription habits, and access to genetic counselling). Furthermore, it should be considered that a complete analysis of an MMR gene in the partner could reveal a VUS making genetic counselling complicated and that comprehensive *PMS2* analysis remains complex with limited availability. The recommendations given here (Rec. 9–11) rely entirely on the Delphi process, which reached a consensus that genetic testing of MMR genes should not be offered to partners of an LS carrier in the absence of consanguinity, the partner belonging to a population with a known founder variant, or the partner having a family history suggestive of LS. However, testing should be offered to the partner of an LS carrier in the presence of any of these three criteria, as well as to partners of CMMRD patients.

### Surveillance

It is widely accepted that CMMRD patients and their parents should be educated about tumour risks associated with CMMRD and about symptoms related to the main tumours. We recommend a clinical examination for children and adults with CMMRD every 6 months and pros and cons of more specific surveillance modalities should be discussed with the CMMRD patient and/or their parents so that they, together with the clinician, can make an informed joint decision to participate in a surveillance program. A greater awareness of being at high risk for developing cancers may increase psychological distress, especially before and after surveillance examinations. In addition, examinations may reveal small lesions of unknown significance in asymptomatic patients for which the only management option is monitoring at a short follow-up interval. Since such situations may increase anxiety, it would be important to provide psycho-oncological support at any time during cancer surveillance and treatment (Table 1d, Rec. 2).

Previous surveillance protocols for CMMRD patients were provided by the C4CMMRD consortium [8], the Pediatric Cancer Working Group of the AACR [29] and the US Multi-Society Task Force on Colorectal Cancer [11]. Observational prospective studies in individuals with CMMRD conducted by the European C4CMMRD consortium and the IRRDC have demonstrated a survival benefit for those individuals with CMMRD who undergo surveillance compared to those who do not [12, 30]. Both studies show that surveillance of the digestive tract and the brain led to early detection of tumours supporting the effectiveness of the suggested surveillance measures [12, 30]. Haematological malignancies were mainly discovered incidentally and between follow-up examinations. Hence, monitoring for haematological malignancies has not proven to be effective. Considering the rapid tumour growth of paediatric NHL, screening requires short intervals between evaluations, which should be performed at least every 3 months. In addition, abdominal ultrasound is not able to detect T-cell lymphoblastic lymphoma, which is the most frequent NHL subtype in CMMRD, located in the mediastinum in most cases. Regular chest X-rays are not recommended for screening because of the potential genotoxic effects of repeated exposure to X-rays. While blood count may be useful in cases of bone marrow infiltration, it has limited value in ALL or NHL surveillance [50]. Although regular blood sampling for detection of circulating T-cell rearrangement may be a potential option for early diagnosis of T-cell lymphoblastic lymphoma, its effectiveness in CMMRD is unknown and needs further evaluation in research studies. Based on these observations and previously discussed considerations [51], we recommend only clinical monitoring every 6 months (Rec. 5) and, in contrast to previous guidelines, do not recommend abdominal ultrasound or systematic blood count (Rec. 12-13).

Both the IRRDC study and the C4CMMRD study evaluating the previous surveillance guidelines support a six-month interval for brain MRI. Before the introduction of surveillance programmes, most CMMRD-patients did not reach adulthood, therefore studies assessing the brain tumour risk in an adult population are not available. Nonetheless, given their already intensive surveillance program, the recommendation to perform annual screening in adults from the age of 20 years by brain imaging achieved consensus (Rec. 8).

All expert groups including the present one agree on endoscopy of the digestive tract as the most effective intervention facilitating early cancer detection and polyp removal before progression into cancer [12]. The progression of adenomas to malignancy in CMMRD is one of the most rapid of any inherited colorectal cancer syndrome [12, 31], and surveillance intervals should, therefore, not exceed 1 year increasing to an approximately 6-months interval once polyps are detected (Rec. 18, 20). Given the diagnoses of small bowel and stomach cancers in CMMRD patients as young as 9 years, it is recommended video capsule endoscopy (VCE) and gastroscopic surveillance are started at the same time as colonoscopy, which is recommended to start at 6 years (Rec. 15, 18, 21). To date there is no data about value of MRI of the small bowel in CMMRD patients but this exam could be considered in combination with push enteroscopy with careful inspection of the ampullary region because small bowel neoplasms are often proximally located and may be missed by VCE ([52], Rec. 16).

The incidence of gynaecological and urinary tract tumours in CMMRD is unknown, especially as the majority of these tumours in CMMRD have been reported at an age that most CMMRD patients do not reach. Expert advice during the Delphi process was to not recommend annual urine cytology and urine dipstick to CMMRD patients because their benefit in LS patients has not been demonstrated (Rec. 24). The same decision was made for endometrial biopsy, which significantly adds to the burden of surveillance by the pain it causes. Instead, it is recommended that abdomino-pelvic ultrasound, clinical examination, and transvaginal ultrasound are performed (Rec. 22, 25).

We considered the current data to be insufficient to draw conclusions on the effectiveness of whole body MRI (WBMRI) [12]. As the tumour spectrum in CMMRD is different, WBMRI may not have the same efficacy as in Li-Fraumeni syndrome, where sarcomas are more frequent [53–55]. Thus, surveillance by WBMRI is included as optional in the recommendations with the recommendation to offer it at least once to detect malformations and low-grade tumours requiring resection or adapted surveillance (Rec. 27–29). In addition, we encourage collecting data on WBMRI so that its benefit in CMMRD can be assessed. As brain tumours are the major oncological risk in CMMRD patients, WBMRI should not replace specific brain imaging.

Based on data of the 64 low-grade tumours detected, Durno and colleagues [12] found that the cumulative likelihood of transformation to high-grade cancer was 81% for gastrointestinal cancers within 8 years and 100% for gliomas within 6 years. Hence, we recommend that resection or specific surveillance of low-grade lesions should be offered to CMMRD patients (Rec 28).

### Clinical management

To date, surgery is still the mainstay for resectable high-grade glioma. It has been shown that recurrent glioblastomas after temozolomide treatment frequently exhibit a hypermutated phenotype with defective MMR [56] confirming previous preclinical evidence that MMR defects are a major mechanism of resistance to temozolomide [38]. Taken together, temozolomide is no longer recommended in CMMRD patients (Rec. 6). Given the high response rate to ICI in patients with CMMRD [35–37], including ICI in the front-line treatment of patients with high grade glioma should be considered (Rec. 7).

Even though MMR deficient cells have been shown to have a certain degree of tolerance to thiopurines in vitro, clinical data obtained in a large series of CMMRD-associated NHL do not demonstrate an increased risk of treatment failure in CMMRD patients treated with the current standard regimens as compared to patients with sporadic NHL [57]. The treatment approach for CMMRD NHL and leukaemia should probably not differ from treatment of sporadic cases (Rec. 10, 12). Also, haematopoietic stem cell transplantation seems feasible [14] (Rec. 5).

ICI have shown their efficacy in MSI tumours and now represent the standard of care for metastatic and advanced colorectal cancer patients with LS [58]. Therefore, it is recommended that management of cancers of the LS spectrum in a CMMRD patient follow treatment guidelines designed for patients with LS associated tumours, and that immunotherapy should be used as front-line treatment of large, unresectable, or metastatic colorectal tumours in a CMMRD patient (Rec. 13-14). Decisions regarding the management of colorectal polyposis in these patients should take several considerations into account. An early and extensive surgical management, such as total colectomy, might appear indicated due to the much faster progression from adenoma to carcinoma in CMMRD than in other adenomatous polyposis syndromes. However, it should also be considered that in CMMRD (i) the presence of multiple polyps is common, but usually in numbers manageable by endoscopic resection, (ii) immunotherapy is an effective treatment option for carcinomas of the digestive tract, (iii) extensive colonic surgery has an adverse impact on quality of life in these young patients, and (iv) the poor prognosis of these patients is mainly due to other, particularly brain, tumours. Taken together, we do not recommend specific and/or early digestive surgical management for CMMRD patients. Even though CMMRD-associated polyps more frequently show high-grade dysplasia, as in other adenomatous polyposis syndromes [59, 60], surgical decisions should be mainly guided by whether or not the polyposis can be controlled endoscopically (Rec 17), considering endoscopic interventions every 6 months if needed (surveillance Rec 18).

A potential preventive effect of immunotherapy or acetylsalicylic acid on polyposis development is uncertain. There is currently insufficient evidence in the literature to recommend the use for chemoprevention, though the potential benefits and side effects of preventive treatment with acetylsalicylic acid should probably be discussed with CMMRD patients (Rec. 22).

Considering the high incidence of multiple tumours in CMMRD patients, we recommend separate sampling and molecular analysis of synchronous tumours as they might be distinct entities. Cancer surveillance should be performed at the time of diagnosis as well as during and after the period of cancer treatment (Rec. 18) and in case of suspicion of a relapse, it is crucial to consider the possibility of a second primary disease rather than a relapse and to perform molecular analysis of samples at initial diagnosis and relapse to make an accurate diagnosis (Rec. 19-20).

### Future research

Being so rare, characterisation of CMMRD pathology and clinical course is difficult and requires concerted, international efforts. Therefore, a general priority for future research is to maintain and expand patient registries as well as the clinical and academic networks around CMMRD.

Currently, there are only a few retrospective studies that can provide empirical evidence for the prevalence of CMMRD among different cancer patient populations (Supp. Tables S1 & S7 in [61]) [62–64]. These studies rely largely on genetic CMMRD diagnosis using high-throughput sequencing methods and are associated with some uncertainties. Prospective screening for CMMRD in relevant patient cohorts (paediatric high-grade glioma or T-lymphoblastic lymphoma patients, children suspected of sporadic NF1, etc.) using scalable, highly reliable, and low-cost ancillary assays [24–27], should improve frequency estimation which will have an impact on future guidelines for CMMRD diagnosis. Although associated with more limitations, tumour-based molecular screening, e.g. by immunohistochemistry analysis of MMR protein expression [65] and analyses of tumour mutation burden and mutational signatures [61, 66], could also be used to identify CMMRD patients in relevant patient cohorts. Such studies would also lay the basis for an evaluation of the sensitivity and specificity of the clinical indication criteria for CMMRD in cancer patients (Table 2) to assess their efficacy and potential weaknesses for refinement.

As has been seen in other cancer syndromes, the ascertainment bias affecting retrospective studies of “familial cases”, if not corrected by dedicated statistical methods, may lead to an over-estimation of cancer risks associated with CMMRD. Molecular screening for CMMRD in cancer patient cohorts recruited with limited clinical and/or familial selection criteria may identify more CMMRD patients and provide less biased estimates of cancer risks associated with CMMRD, which may have implications for future diagnosis and management guidelines. Further exploration of MMR genotype-phenotype correlations including characterization of hypomorphic variants [6, 7] could facilitate better risk stratification, provide general insight into MMR function, and tailor future recommendations to patients’ genotypes.

Although cancer surveillance in CMMRD has been shown to improve survival [12, 30], further assessment of its efficacy and impact is needed. In particular, evaluation of the clinical utility of WBMRI in prospective studies is needed. The improved overall survival of CMMRD patients due to surveillance and therapeutic advancements would mean that more patients will reach an older age. This has implications for both our understanding of the CMMRD phenotype and its management. In particular, the CMMRD cancer spectrum may change with age and surveillance protocols may need to be adapted accordingly. Studies on the acceptability and psychological impact of, as well as long-term adherence to surveillance interventions are currently lacking.

CMMRD cancer risk could also be managed through prevention. Immune-based prophylaxis may provide a novel approach to CMMRD cancer prevention. Vaccines have been developed based on frameshift peptides associated with MMR deficient cancers in humans, and have been shown to be safe in a phase I/IIa clinical trial [67] and to induce an immune response [67, 68]. Therefore, vaccination of CMMRD patients with frameshift peptide neoantigens could be trialled in the future. Alternatively, daily acetylsalicylic acid intake approximately halved the incidence of colorectal carcinomas in LS carriers in the CAPP2 randomised control trial [69] and could be beneficial to CMMRD patients [70]. However, there is currently no strong evidence to support a reduction of cancer incidence by acetylsalicylic acid or other non-steroidal anti-inflammatory drugs in CMMRD, and further studies are needed.

The loss of MMR function in CMMRD tumours makes them resistant to certain chemotherapies, whilst sensitising them to ICI. CMMRD tumours, therefore, may require specific management. Mechanisms of therapy resistance also require exploration. Research in this area is ongoing and our understanding is rapidly changing. Promising biomarkers for ICI response include TMB, with higher TMB being predictive of positive ICI response among MMR deficient metastatic colorectal carcinomas [71], and measures of intra-tumoural immune activity, such as Immunoscore® [72]. Studies of biomarkers of ICI response and resistance in the context of different CMMRD tumours are needed.

Studies of CMMRD tumourigenesis pathways will improve our understanding of the CMMRD phenotype and allow us to optimise management. Systematic biobanking of neoplastic tissues will be particularly useful to answer these questions. Model systems, such as cell lines, organoids, and mice, will also be helpful.

## Conclusion

Although these guidelines are written primarily for geneticists and paediatric haemato-oncologists, they can also be used by other physicians, patients or other interested parties. The recommendations given here are statements to support decision making, based on systematically evaluated evidence for a specified clinical setting. Whilst these clinical guidelines are based on the latest published evidence, care of each individual remains primarily the responsibility of their treating medical professionals. Decisions for care should always be based on the needs, preferences and circumstances of each patient. These clinical guidelines should support clinical decision making, but never replace clinical professionals.
